# Correction: Kim et al. Multi-Threaded Sound Propagation Algorithm to Improve Performance on Mobile Devices. *Sensors* 2023, *23*, 973

**DOI:** 10.3390/s26113579

**Published:** 2026-06-04

**Authors:** Eunjae Kim, Sukwon Choi, Cheong Ghil Kim, Woo-Chan Park

**Affiliations:** 1 Department of Computer Science and Engineering, Sejong University, Seoul 05006, Republic of Korea; 2 Department of Computer Science, Namseoul University, Cheonan 31020, Republic of Korea

**Text Correction**

In the original publication [1], references [1,2] were identified as unrelated or inappropriate references to the study. Therefore, a correction should be made to Section 1, Introduction. The sentence “Recently, as interest in blockchain/metaverse/XR/VR/MR has increased [1,2], more research to improve the sense of reality and immersion has been conducted” should be replaced by “Recently, as interest in blockchain/metaverse/XR/VR/MR has increased, more research to improve the sense of reality and immersion has been conducted”.

**References** **Correction**

The citations of references [1,2] should be removed. With this correction, the order of some references has been adjusted accordingly.

The authors state that the scientific conclusions are unaffected. This correction was approved by the Academic Editor. The original publication has also been updated.
